# Supplementary material for: Structure and function of Plasmodium actin II in the parasite mosquito stages
Source: PLoS Pathog. 2023 Mar 6;19(3):e1011174. doi: 10.1371/journal.ppat.1011174 (PMC10019781; doi:10.1371/journal.ppat.1011174)
Supplement: S1 Table — (DOCX) [file ppat.1011174.s001.docx]

**S1 Table.** Data collection and refinement statistics.

| **Data collection** | **Actin II** | **Actin II-JAS** |
| --- | --- | --- |
| Magnification | 75000x | 75000x |
| Defocus range (mm) -Voltage (kV) | -300 | -300 |
| Microscope | Titan Krios | Titan Krios |
| Detector | Falcon 3 | Falcon 3 |
| No. of frames | 46 | 46 |
| Pixel size (Å/pixel) | 1.09 | 1.09 |
| No. of micrographs | 1058 | 3977 |
| **Reconstruction (Relion 3.1beta)** |  |  |
| No. of helical segments | 47 197 | 272 310 |
| Box size (px) | 328 | 328 |
| Rise (Å) | 28.34 | 28.37 |
| Azimuthal rotation (°) | -166.9 | -166.9 |
| Average resolution (Å) (FSC=0.143) | 3.5 | 3.3 |
| Model resolution (Å) (FSC=0.5) | 3.4 | 3.2 |
| Map sharpening B-factor (Å^2^) | -111 | -75 |
| **Model building (Phenix 1-19.2-4158)** |  |  |
| No. of chains | 6 | 6 |
| No. of atoms | 18102 | 18324 |
| No. of amino acid residues | 2232 | 2232 |
| No. of ligand atoms (Mg^2+^, ADP, JAS) | 12 | 18 |
| Average B-factor (Å^2^) | 49.25 | 38.63 |
| Average B-factor for ligand atoms (Å^2^) | 37.50 | 46.79 |
| R.m.s.d. bond lengths (Å) | 0.002 | 0.002 |
| R.m.s.d. bond angles (°) | 0.543 | 0.558 |
| CC volume | 0.83 | 0.82 |
| CC masked | 0.85 | 0.84 |
| Molprobity score | 1.17 | 1.12 |
| Clash score | 3.52 | 3.32 |
| Ramachandran plot favored/allowed/outliers (%) | 97.87/2.13/0 | 98.09/1.91/0 |
| Rama-Z (whole/helix/sheet/loop) | 0.45/1.43/0.18/0.65 | 0.64/1.24/0.76/0.43 |
| **Deposition codes** |  |  |
| PDB | 8CCN | 8CCO |
| EMDB | EMD-10588 | EMD-10589 |
