# Supplementary material for: Structure and function of Plasmodium actin II in the parasite mosquito stages
Source: PLoS Pathog. 2023 Mar 6;19(3):e1011174. doi: 10.1371/journal.ppat.1011174 (PMC10019781; doi:10.1371/journal.ppat.1011174)
Supplement: S2 Table — All values are reported as mean ± standard deviation (actin II seeds: n = 3 NP, n = 5 in SS). * Relative k- calculated using the steady state Cc and relative elongation constant (k+) from slope 2 of nucleated polymerization assays. Raw data related to the table can be found in S7 Data. (DOCX) [file ppat.1011174.s002.docx]

**S2 Table.** Relative kinetic parameters of actin II polymerization. All values are reported as mean ± standard deviation (actin II seeds: n=3 NP, n= 5 in SS). * Relative k_-_ calculated using the steady state C_c_ and relative elongation constant (k_+_) from slope 2 of nucleated polymerization assays. Raw data related to the table can be found in S7 Data.

| **Nucleated polymerization (NP)** | | | |
| --- | --- | --- | --- |
|  | **Actin II seeds** | | |
|  | *Slope 1* | | *Slope 2* |
| k_+_ (s^-1^) | 0.04 ± 0.01 | | 0.01 ± 0.002 |
| C_c_ (𝜇M) | 1.12 ± 0.29 | | 0.36 ± 0.19 |
| k_-_ (s^-1^) | -0.04 ± 0.02 | | -0.004 ± 0.003 |
| **Steady state (SS)** | | | |
| C_c_ (𝜇M) | | 0.11 ± 0.02 | |
| k_-_ (s^-1^)**^*^** | | -0.003 ± 2e-004 | |
