# Supplementary material for: Structure and function of Plasmodium actin II in the parasite mosquito stages
Source: PLoS Pathog. 2023 Mar 6;19(3):e1011174. doi: 10.1371/journal.ppat.1011174 (PMC10019781; doi:10.1371/journal.ppat.1011174)
Supplement: S3 Table — (DOCX) [file ppat.1011174.s003.docx]

**S3 Table.** Primers used for DNA constructs and genotyping.

|  | **name** | **target** | **sequence** | **restriction site** |
| --- | --- | --- | --- | --- |
| *flag::actinII* construct | A2CF | actin2 5'FR | GGGGTACCTTTCCCCCATGTTTATTTTA | KpnI |
|  | A2SW5R | actin2 5'FR | GCTAGCCAATTGTGAACACAGATATTATGC | NheI |
|  | A2SW3F | actin2 3'FR | GCGGCCGCAAATGAAGAGATAAATGAGGAG | NotI |
|  | A2CRECO | actin2 3'FR | GGAATTCGATGCATTTGTGTTTGTCTG | EcoRI |
| Genotyping transfected parasites | A2F1 | actin2 5'FR | GGTACCTTTTGAAAACTAACATATTTCTCG |  |
|  | mCherryR | mCherry | CGGAGCCCTCCATGTGAAC |  |
|  | A2F2 | actin2 5'FR | CGCTTGTGGTAAATTGTTATTTCG |  |
|  | A2R | actin2 | ACCCTCATAAATTGGAACAGTG |  |
|  | DHFR | PbDHFR 3'FR | CCATCGATGTTTTTCTTACTTATATATTTATAC |  |
