# Supplementary figures and images for: Structure and function of Plasmodium actin II in the parasite mosquito stages
Source: PLoS Pathog. 2023 Mar 6;19(3):e1011174. doi: 10.1371/journal.ppat.1011174 (PMC10019781; doi:10.1371/journal.ppat.1011174)

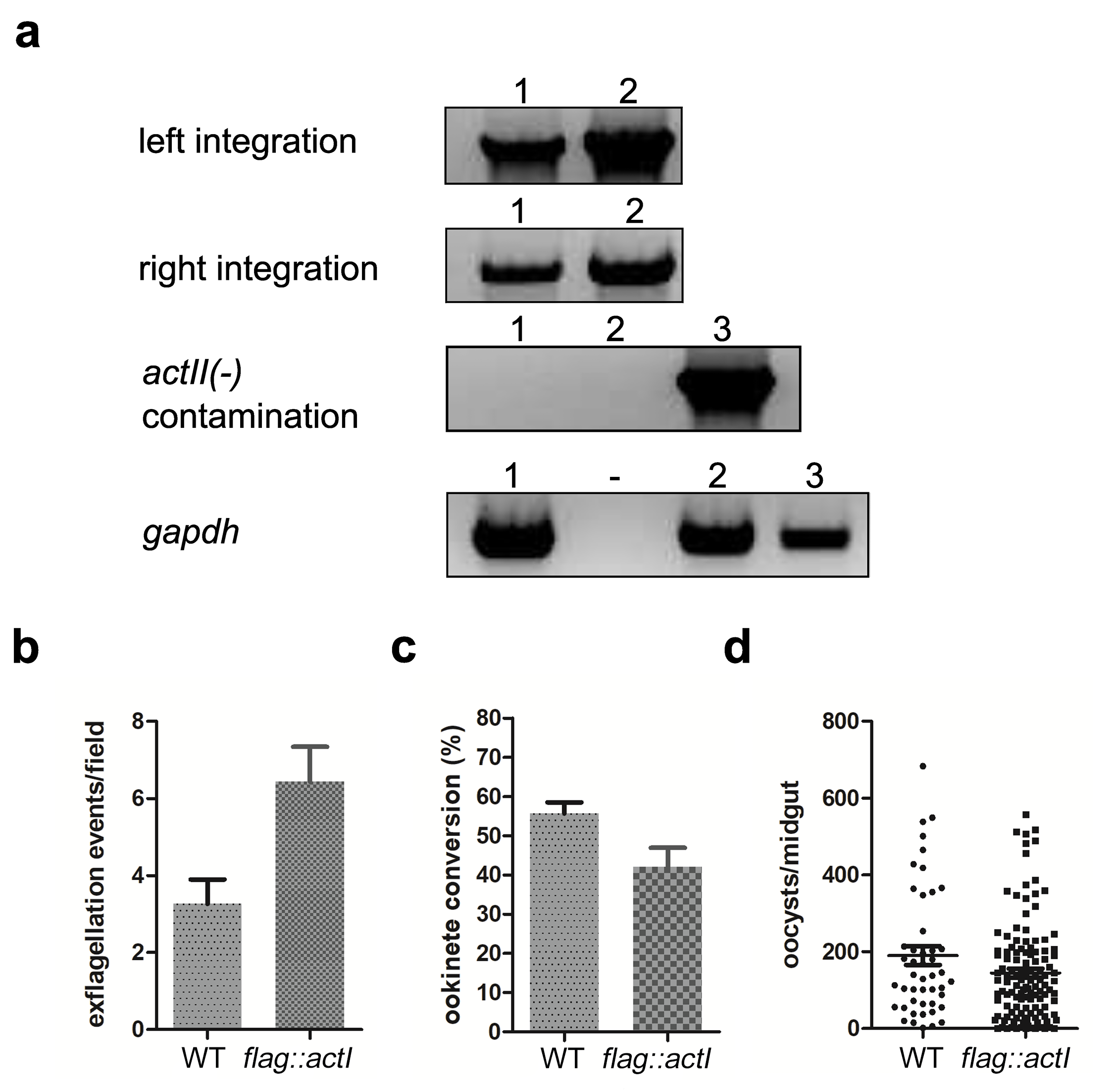

Supplement: S1 Fig — (a) Genotyping flag-actII cloned line. Left integration of the construct was verified with primer pair A2F2 and A2R and right integration with the primer pair DHFR and mCherryR. To control for absence of the actII(-) parasites the primer pair A2F1 and mCherryR was used. Lane 1 and 2: flag::actII, lane 1, 135 ng, lane 2, 50 ng template; lane 3: actII(-). Quality control of gDNA used the gapdh primer pair of the same samples. (b). (b-e) Phenoptyic analysis of flag::actII compared to WT. (b) Exflagellation analysis; average of three experiments of the WT and four of the mutant. (c) Ookinete conversion; three experiments of each strain. Error bars in (b) and (c) are S.E.M. (d) Number of oocysts/midgut. The oocysts were stained with Cap380 antibody and all oocysts irrespective of size are plotted. Differences in (b-d) are not significant, Student’s t-test for (b) and (c), Mann-Whitney for (d) and (e). Raw data related to panels b-d can be found in S8 Data. (TIF) [file ppat.1011174.s004.tif]

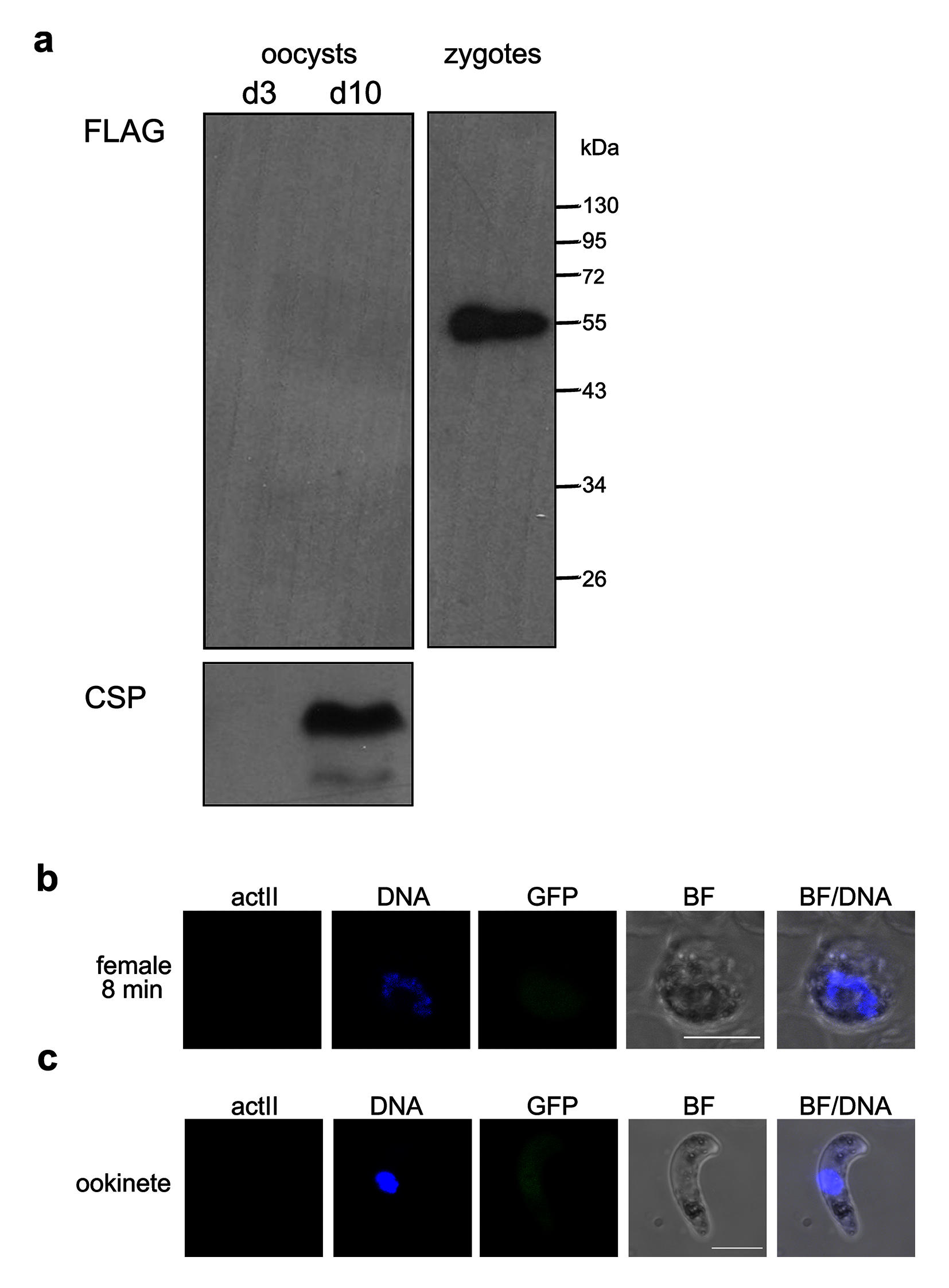

Supplement: S2 Fig — (a) Western blot of extracts from midguts infected with the line expressing FLAG::actinII. Midguts were dissected on day 3 and 10 post blood feeding and crude extracts of 20 and 13 midguts, respectively, were loaded in each lane. Right panel is a positive control with zygote extracts. The samples were run on the same gel and the blot was probed with the anti-FLAG antibody. A duplicate blot was probed with anti-CSP antibody as a loading control; it only gave a signal for the day 10 sample. (b,c) Immunolabeling of flag::actII female gamete 8 min p.a. (b) and ookinete (c). No signal was detected with the anti-FLAG antibody. The background GFP signal (green) is constitutively expressed in this line. DNA was stained with Hoechst 33342 (blue). Scale bars, 5 μm. The female gamete originates from the same experiment as the 8 min sample of male gametes in Fig 1 (lower panels). (TIF) [file ppat.1011174.s005.tif]

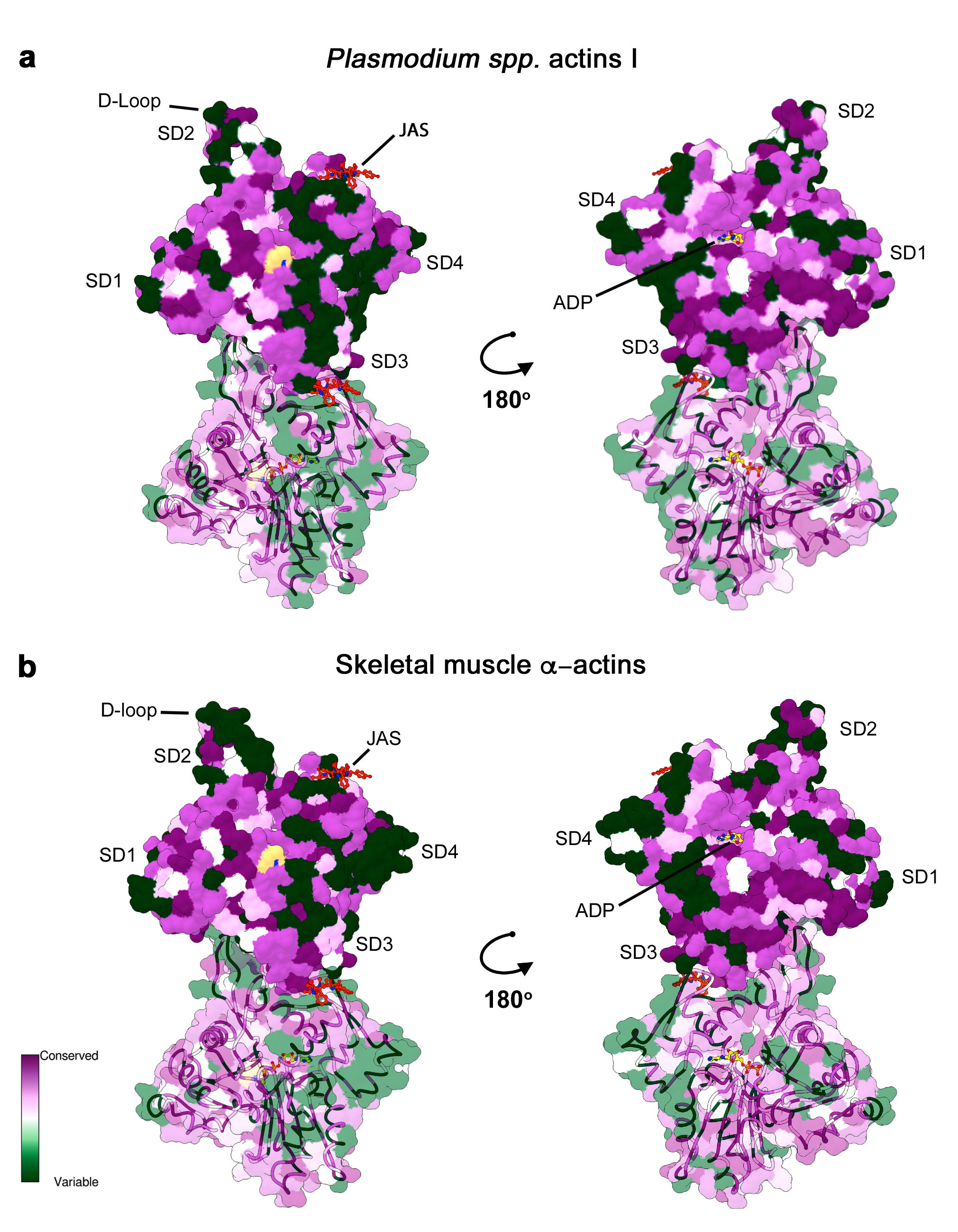

Supplement: S3 Fig — Evolutionary conservation of P. falciparum actin II in comparison with (a) actin I from Plasmodium spp. and (b) skeletal muscle α-actins. The actin surface is colored according to conservation scores; high (purple) to low (green). Amino acid conservation was estimated using the ConSurf server [75]. (TIF) [file ppat.1011174.s006.tif]

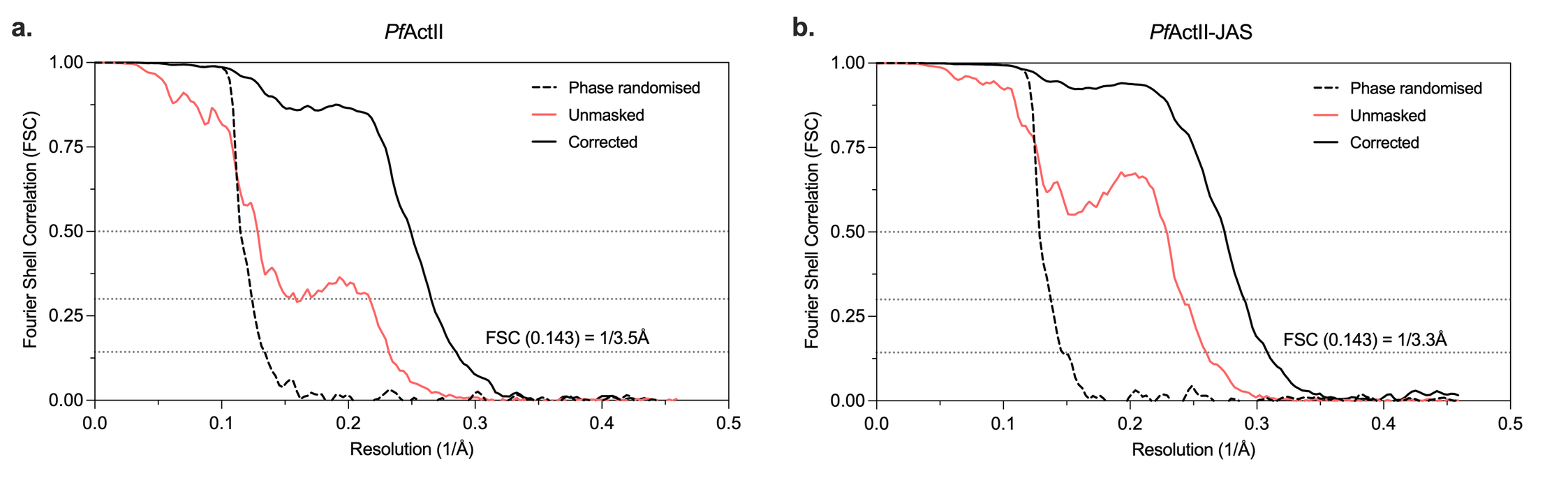

Supplement: S4 Fig — Fourier shell correlation plot of actin II and JAS-stabilized actin II. The corrected curve was calculated from independently refined half-datasets with a soft mask filtered to 15 Å in Relion [76]. (TIF) [file ppat.1011174.s007.tif]

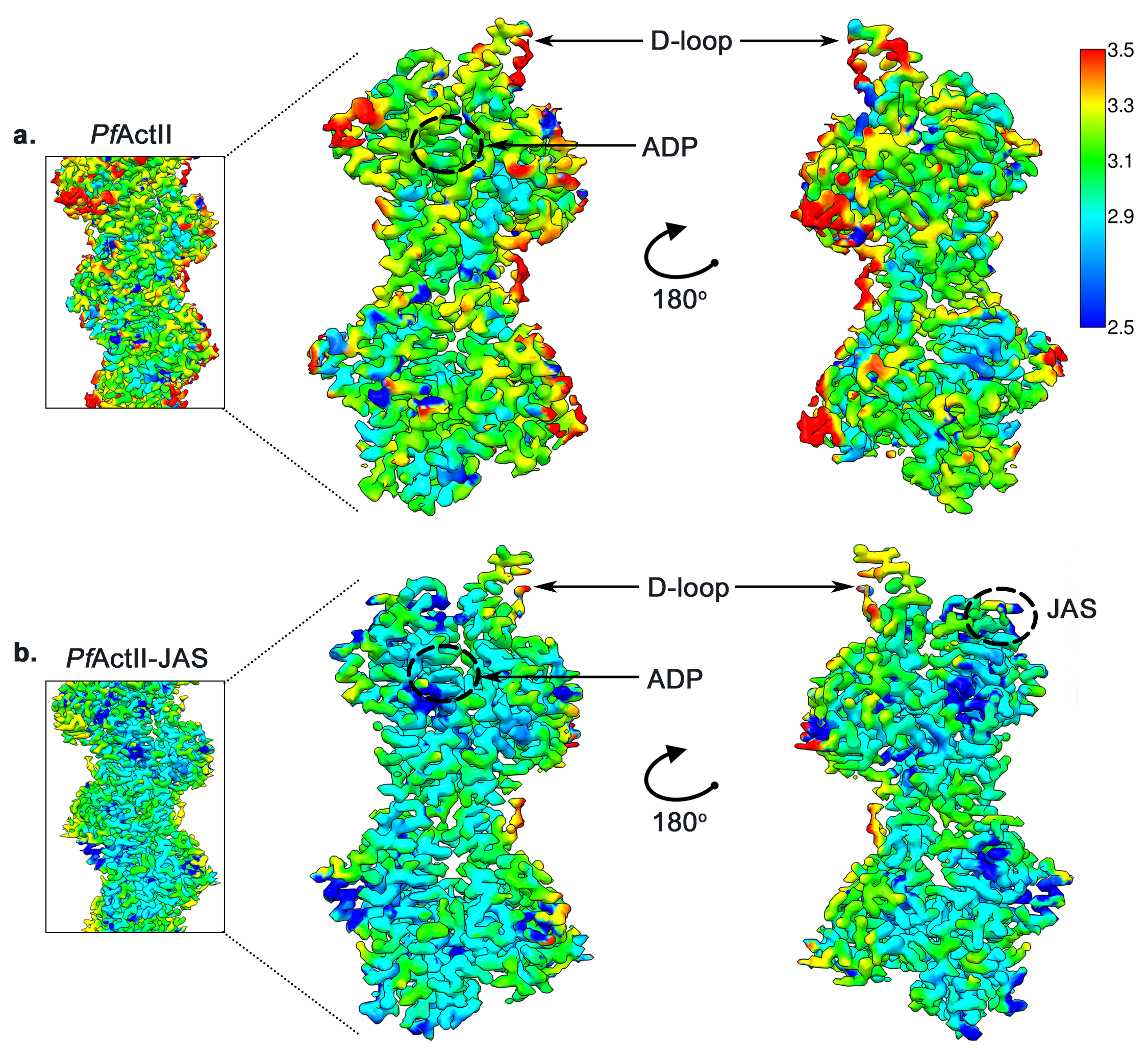

Supplement: S5 Fig — Local resolution of actin II (a) and JAS-stabilized actin II (b). The local resolution estimation is based on Fourier shell correlation threshold 0.143 calculated with Blocres in the Bsoft software package, applied to the final sharpened map. The left panel shows a central section of the filament. On the right, two adjacent protomers are shown and, the ligands densities are highlighted [68]. (TIF) [file ppat.1011174.s008.tif]

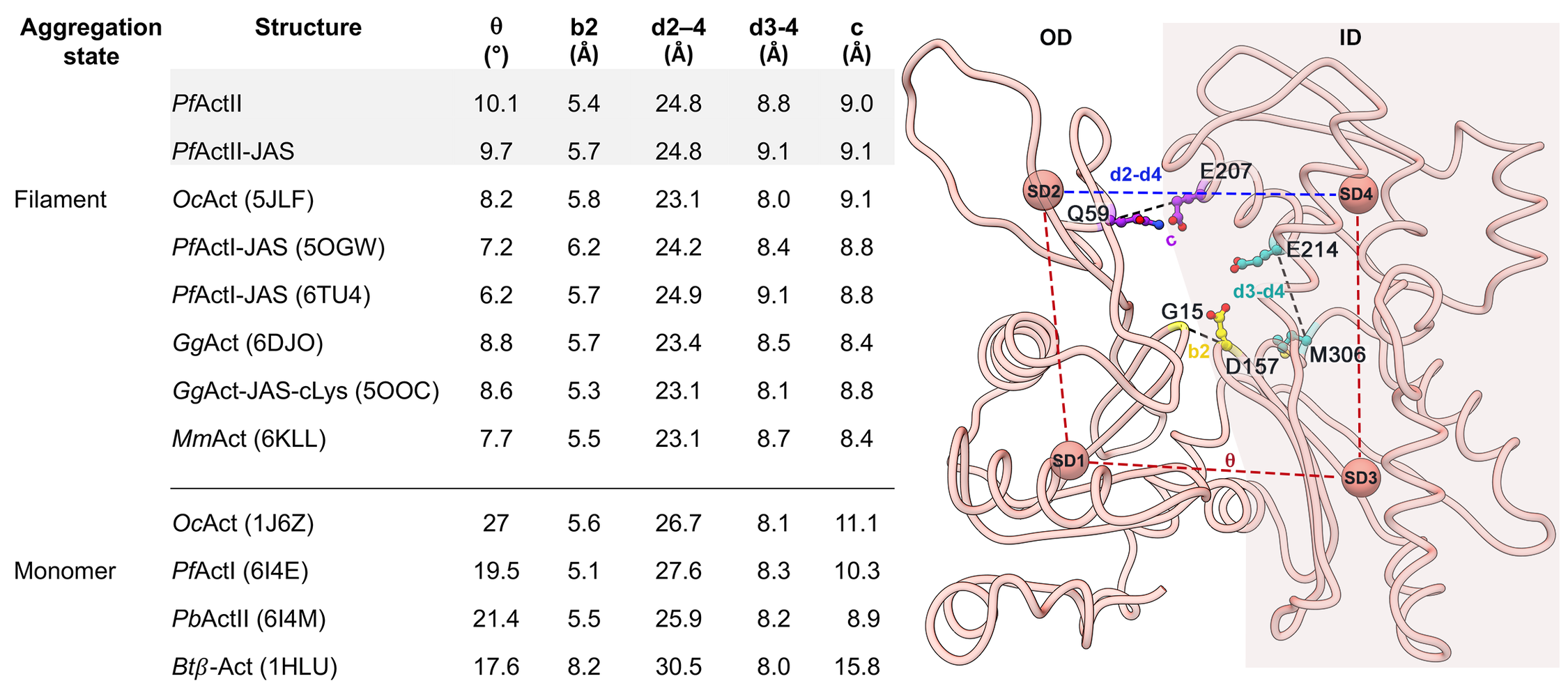

Supplement: S6 Fig — The distance of the twist angles of the centers of mass of SDs (θ), the phosphate clamp distance (b2), the distance of SD1 and SD2 (d2-4), SD3-SD4 (d3-4), and cleft mouth (c) calculated for different actin structures. The dihedral angle of subdomains in the JAS-stabilized actin II model is rotated 11.8° relative to the crystal structure of G-actin II-gelsolin (PDB: 6I4M), 2.4° relative to JAS stabilized F-actin I (5OGW), and 3.4° relative to the JAS stabilized F-actin I in complex with myosin A (6TU4). (TIF) [file ppat.1011174.s009.tif]

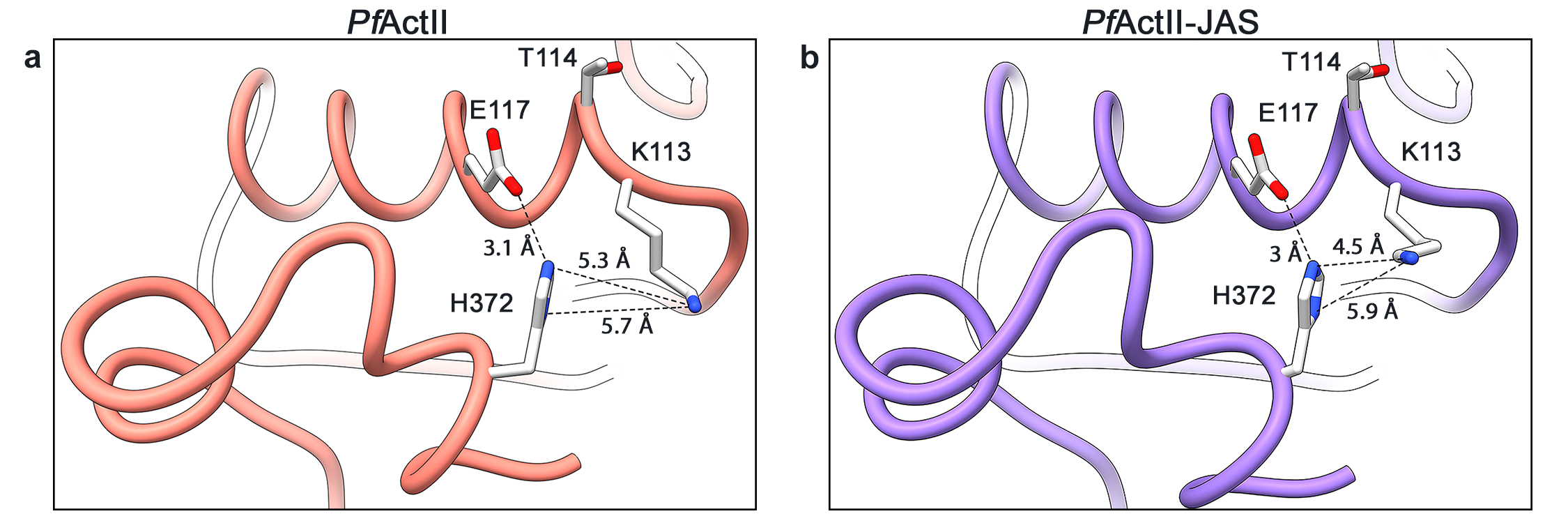

Supplement: S7 Fig — (a) In F-actin II, the ring of H372 is oriented towards the E117. (b) In F-actin II, the N1 of the H372 turns towards K113. The distance between H372 and K113 is more than 5 Å in both structures. (TIF) [file ppat.1011174.s010.tif]

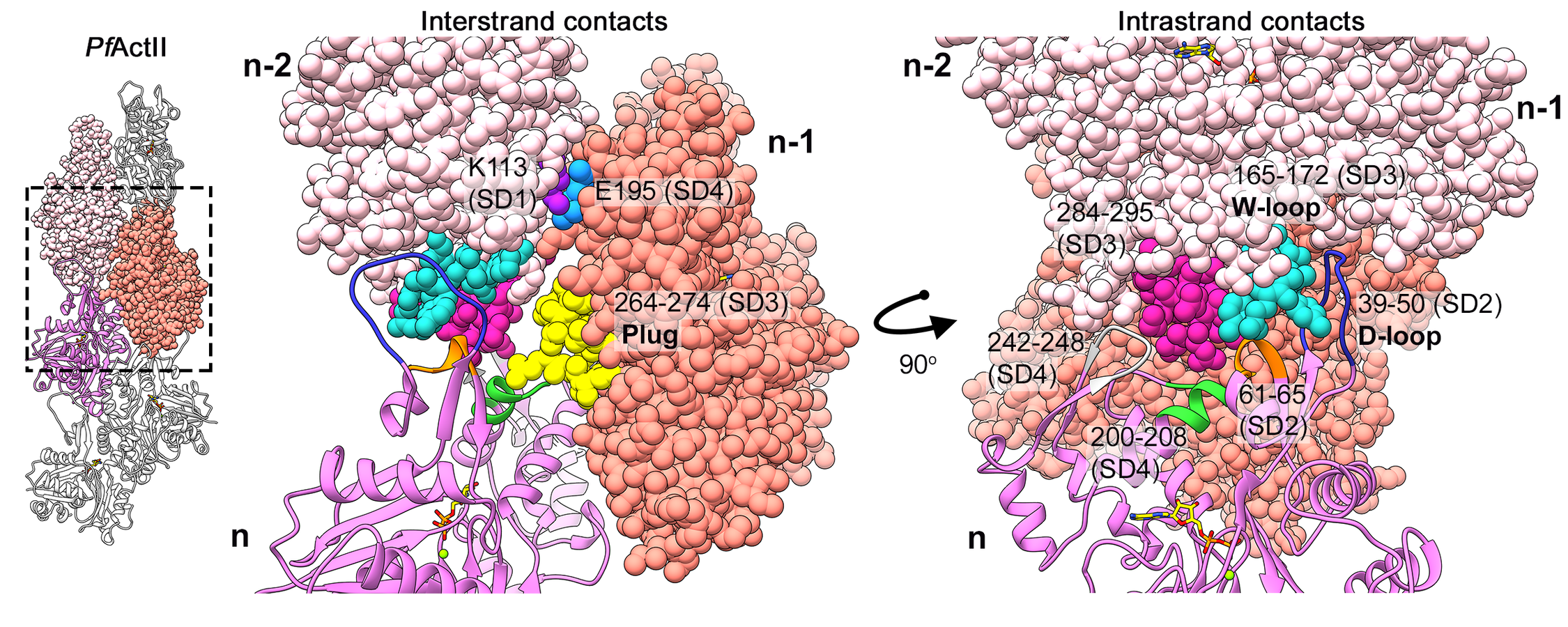

Supplement: S8 Fig — Two protomers are represented by spheres n-1 and n-2 (salmon and pink); the silhouette of the third protomer (n) is visualized by ribbon representation in Chimera. (TIF) [file ppat.1011174.s011.tif]

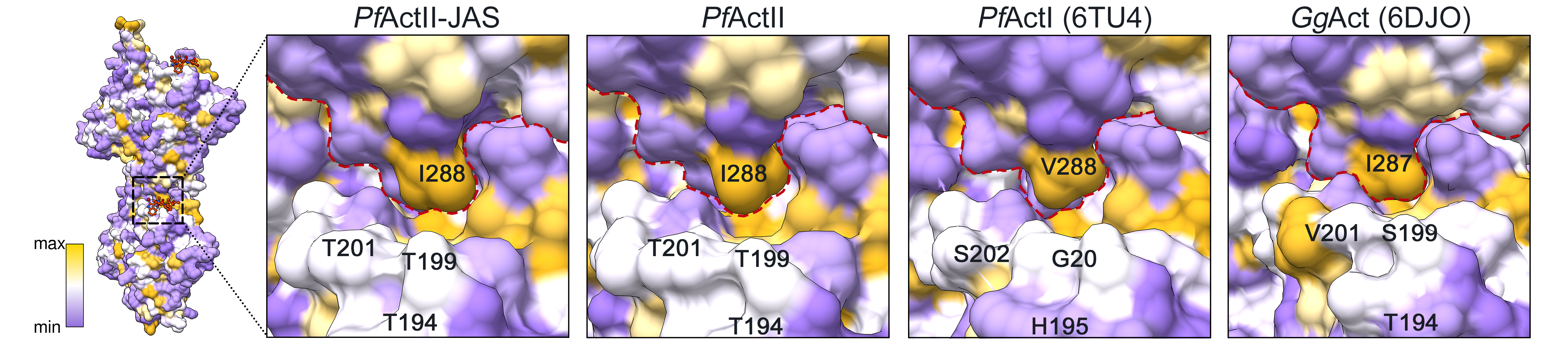

Supplement: S9 Fig — (a) P. falciparum F-actin II, (b) JAS-stabilized F-actin II, (c) P. falciparum F-actin I (6TU4), (d) filamentous skeletal muscle α-actin (6DJO). In actin II, I288 inserts into a groove in the adjacent protomer, resembling a lock-key interaction, like in canonical actins. The actin surface is colored according to hydrophobicity; high (yellow), medium (white), and low (purple). (TIF) [file ppat.1011174.s012.tif]

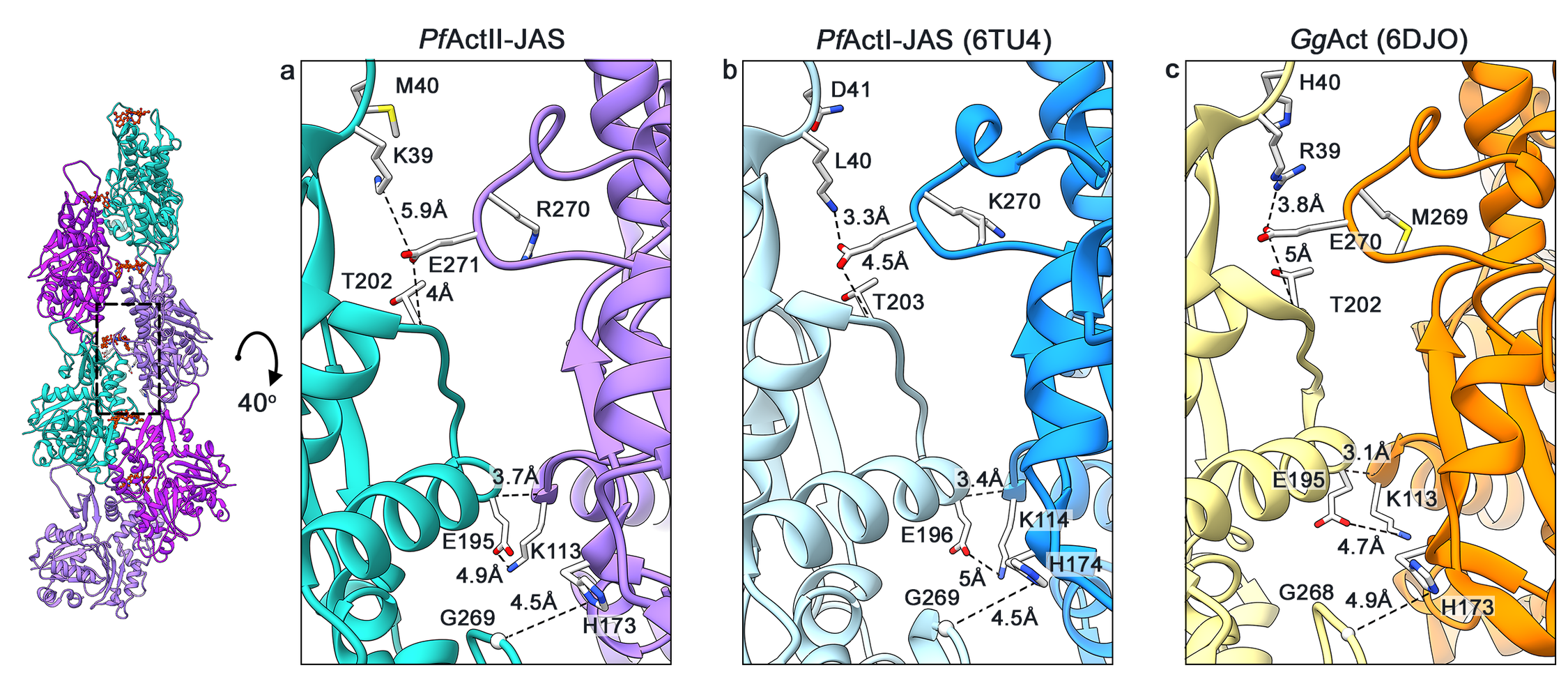

Supplement: S10 Fig — Lateral contacts of protomers from a different strand of (a) F-actin II-JAS, (b) F-actin I (6TU4), (c) O. cuniculus F-actin (6DJO). Distances between the residues are indicated in black dashed lines. (TIF) [file ppat.1011174.s013.tif]

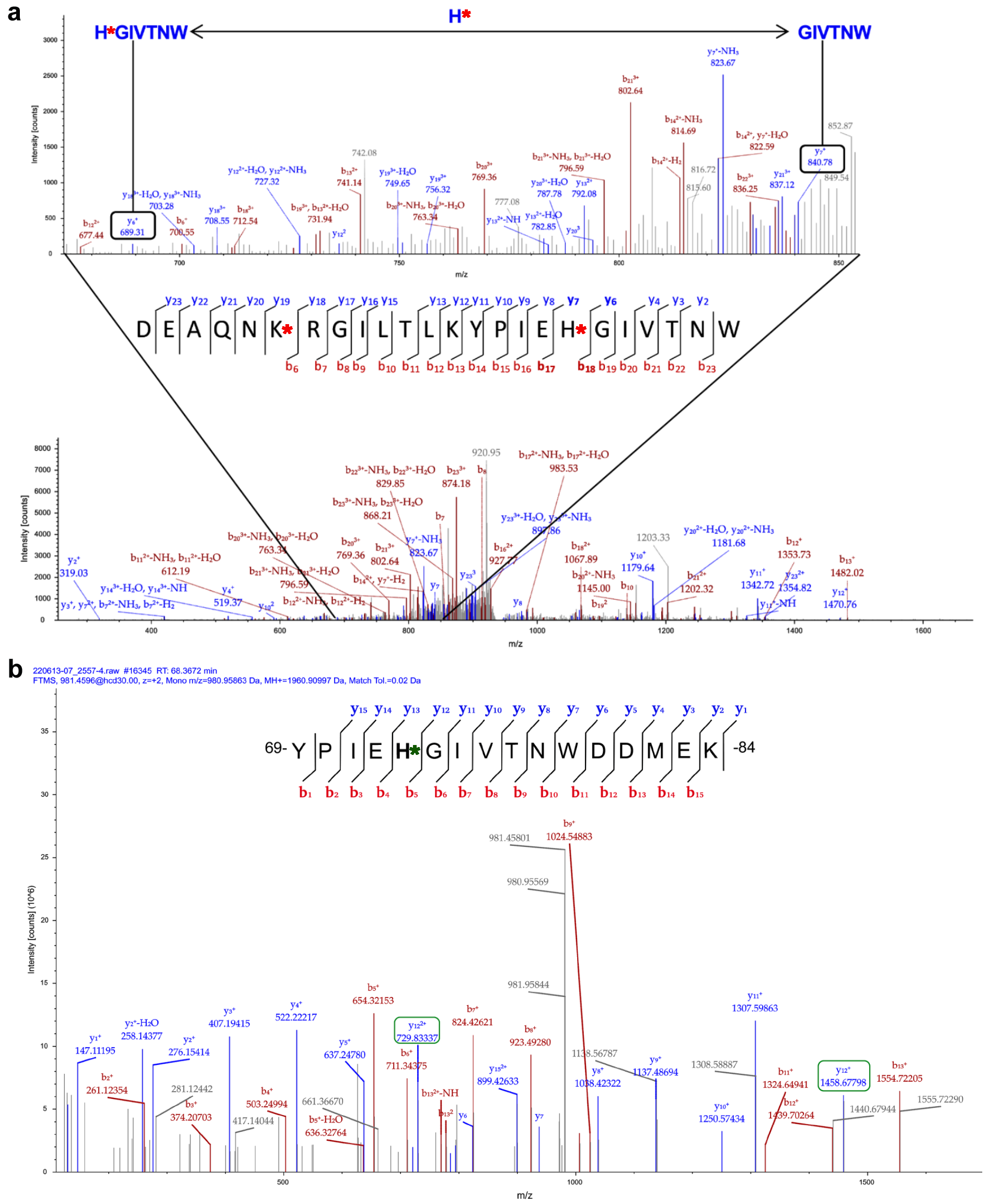

Supplement: S11 Fig — (a) PbActII peptide 56–79. The red stars indicate methylated residues. (b) Recombinant PfActII peptide 69–84. Methylated H73 is indicated by a green star. Methylated double and triple-charged signals are highlighted by green boxes. The b and y series ions detected are shown in red and blue, respectively, in both panels. The m/z difference between these ions corresponds to a methylated histidine. Raw data related to this figure can be found in S9 Data. (TIF) [file ppat.1011174.s014.tif]

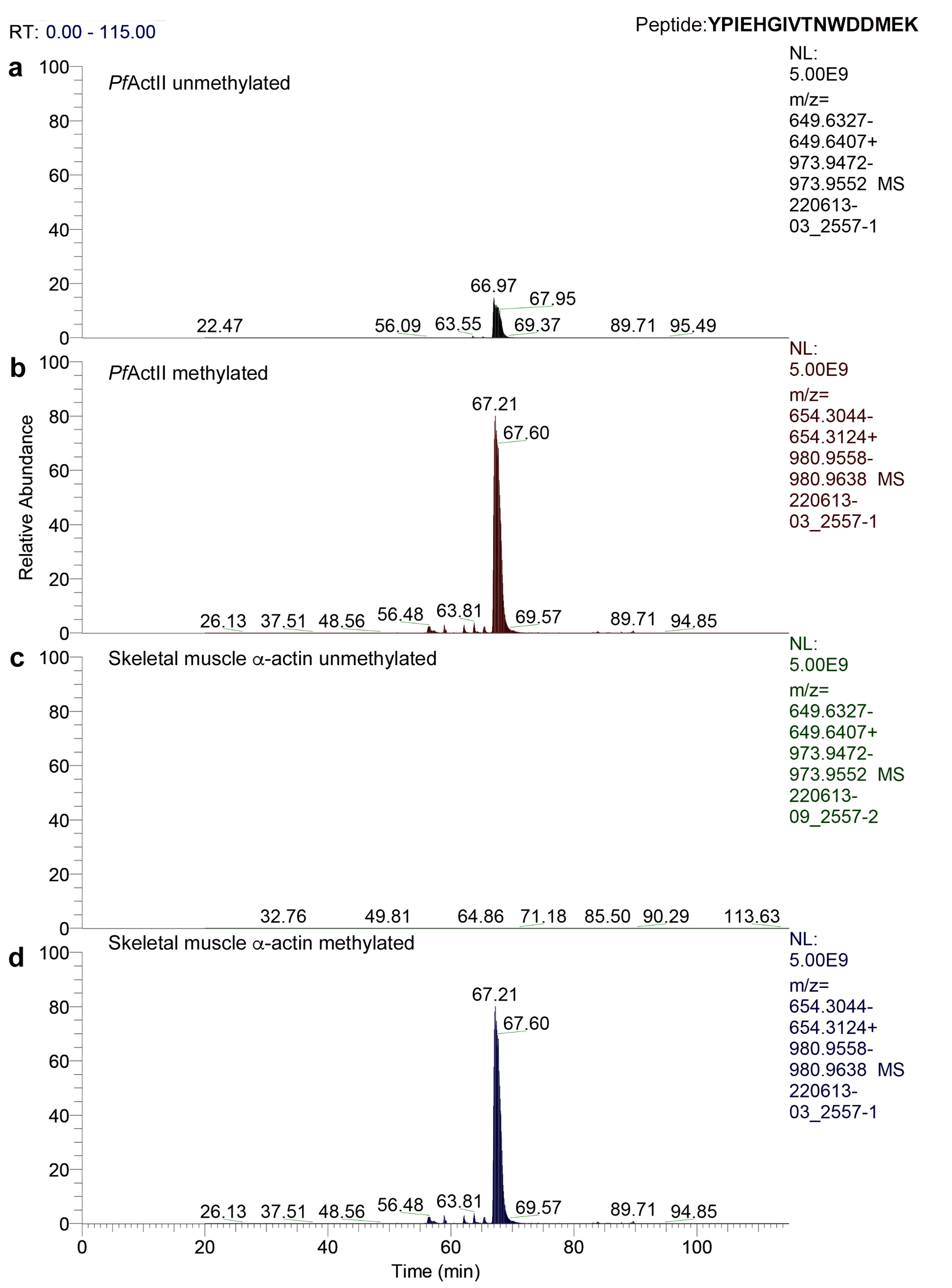

Supplement: S12 Fig — (a) Unmethylated and (b) methylated PfActII. (c) Unmethylated and (d) methylated skeletal muscle α-actin. Raw data related to this figure can be found in S9 Data. (TIF) [file ppat.1011174.s015.tif]

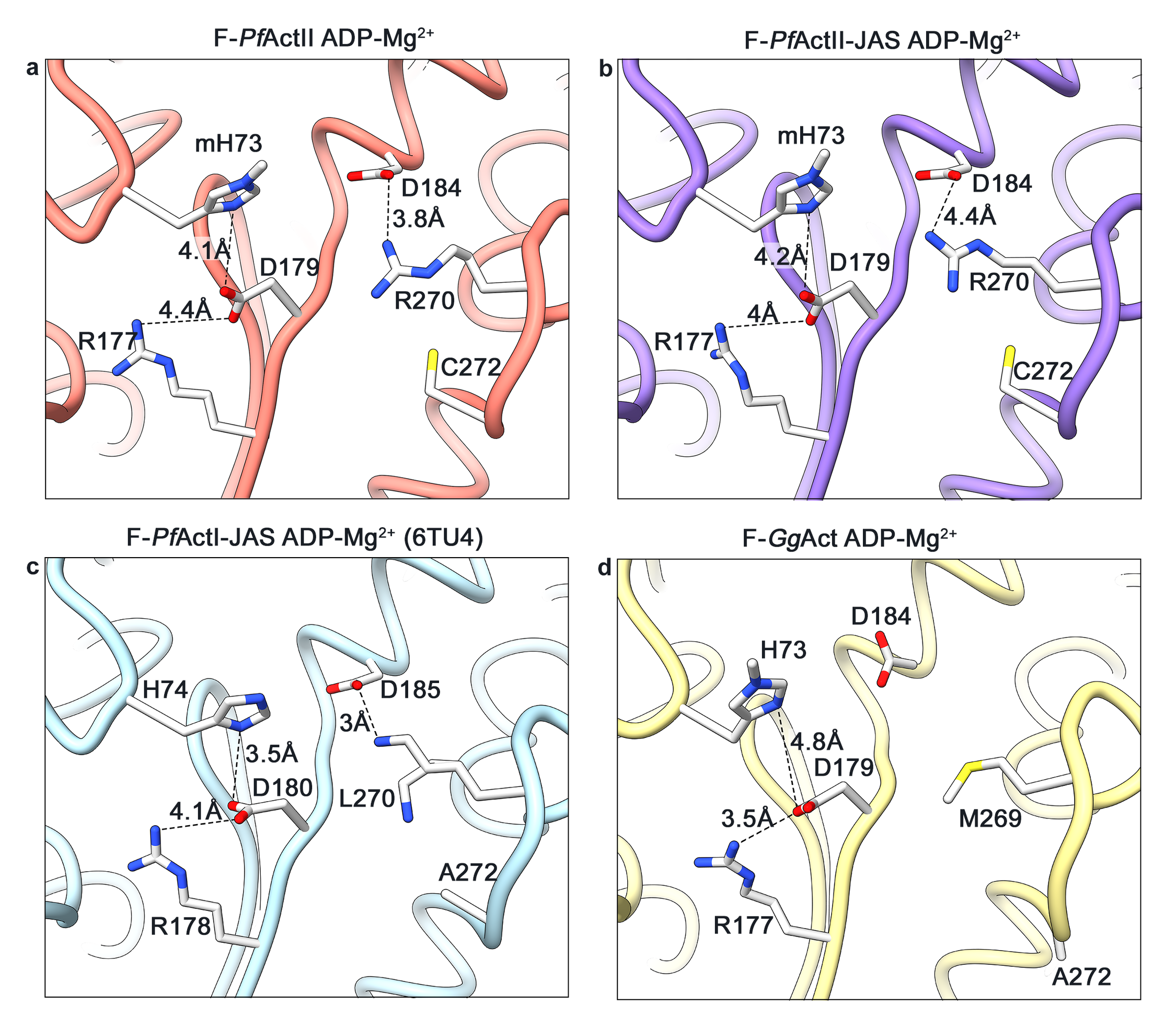

Supplement: S13 Fig — (a) F-actin II, (b) F-actin II-JAS, (c) F-actin I-JAS (6TU4) and (d) G. gallus F-actin (6DJO). All structures are in the Mg-ADP state and show a 1b conformation of the A-loop. The most probable ionic and hydrogen bonds are indicated with dashed lines. (TIF) [file ppat.1011174.s016.tif]

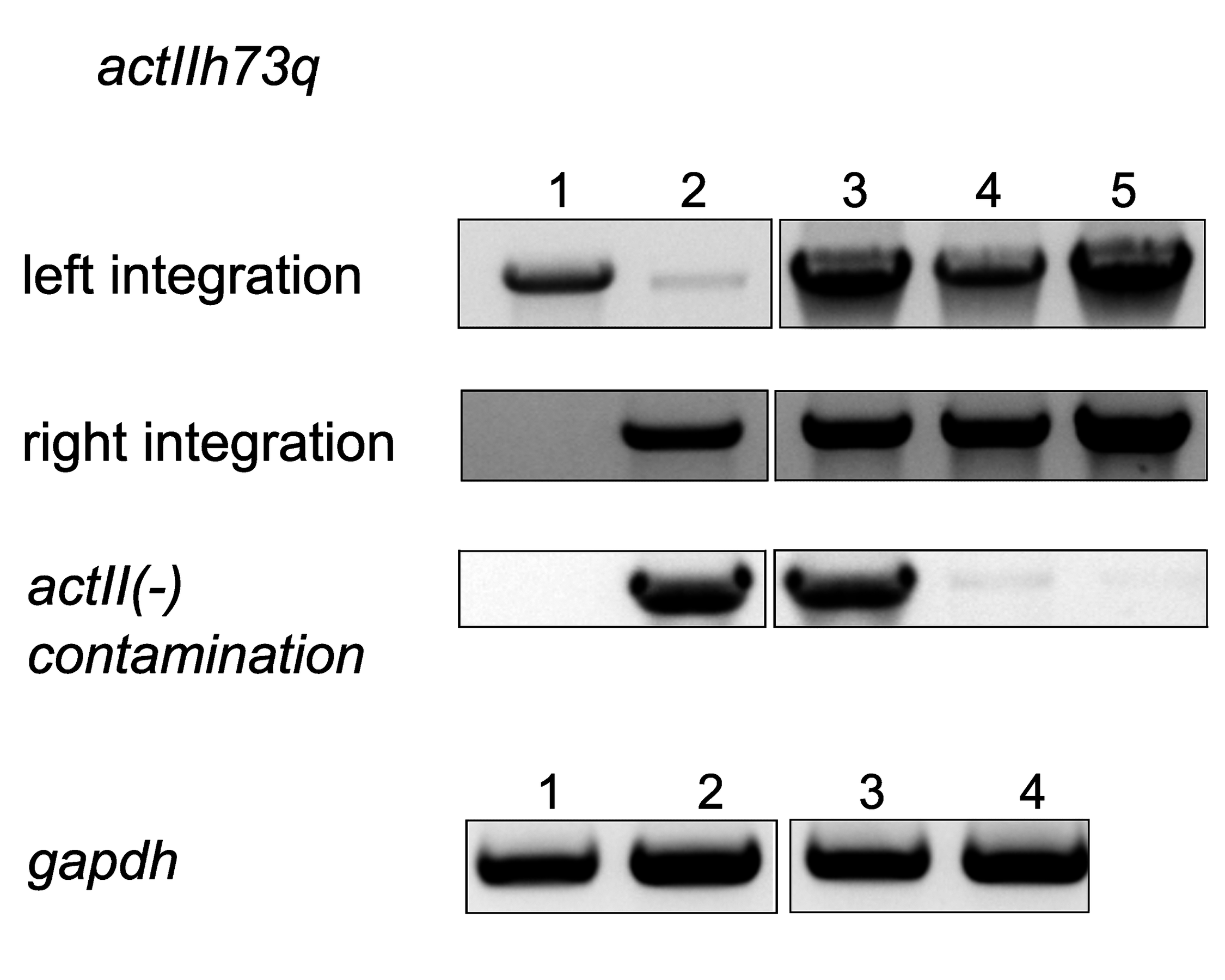

Supplement: S14 Fig — To control for absence of the actII(-) parasites the primer pair A2F1 and mCherryR was used. 1: WT; 2: positive control; 3: transfected parasites with actIIh73q construct; 4 and 5: actIIh73q clone 1. For quality control of the gDNA GAPDH primer pair was used. (TIF) [file ppat.1011174.s017.tif]

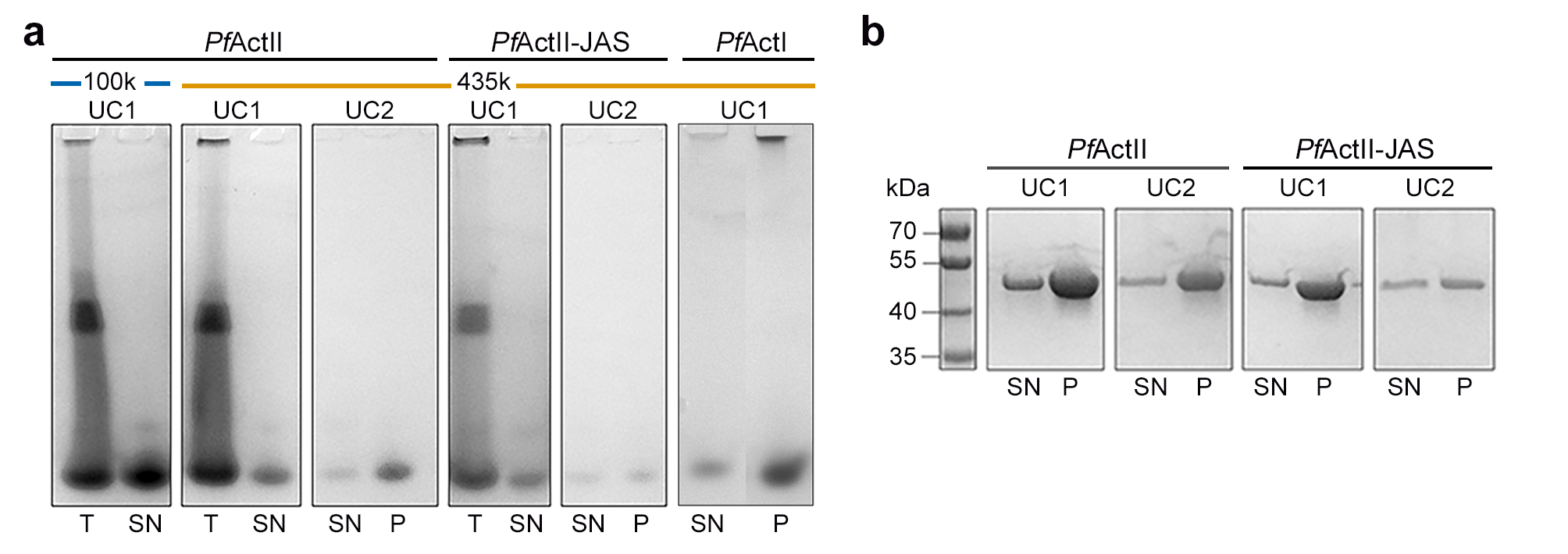

Supplement: S15 Fig — (a) Native PAGE of Plasmodium actins. Samples were polymerized overnight, pellet (P) and supernatant (SN) were separated by ultracentrifugation at 4°C for 1 h (UC1). The SN fraction was re-pelleted 16 h after the initial ultracentrifugation (UC2). (b) SDS-PAGE of the two steps of ultracentrifugation at 435000 g of actin II. (TIF) [file ppat.1011174.s018.tif]

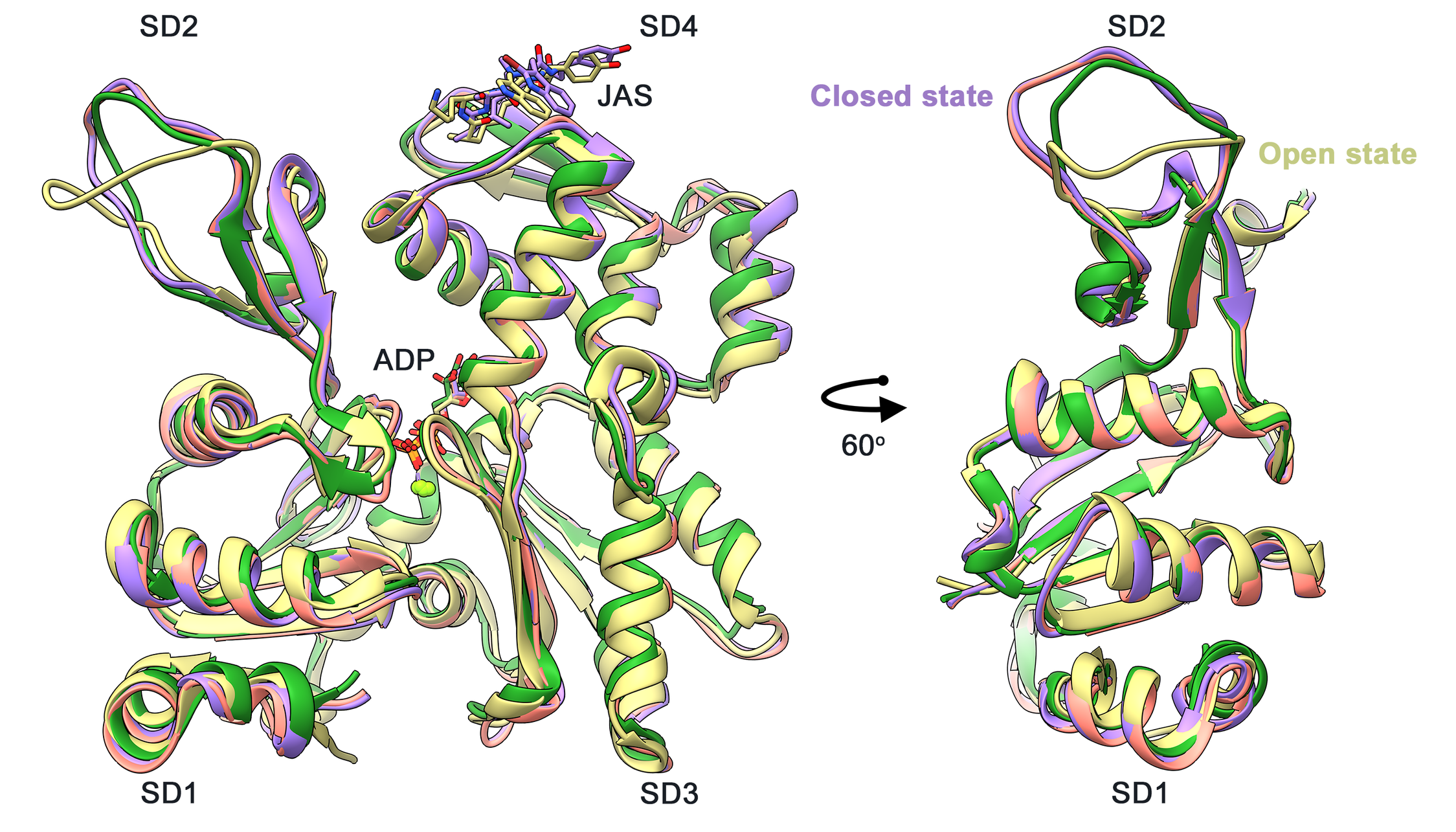

Supplement: S16 Fig — cuniculus F-actin (5OOC), and O. cuniculus F-actin (5ONV). JAS is not altering the conformation of the D-loop of actin II like in actin from O. cuniculus, in which the D-loop has an open conformation. (TIF) [file ppat.1011174.s019.tif]

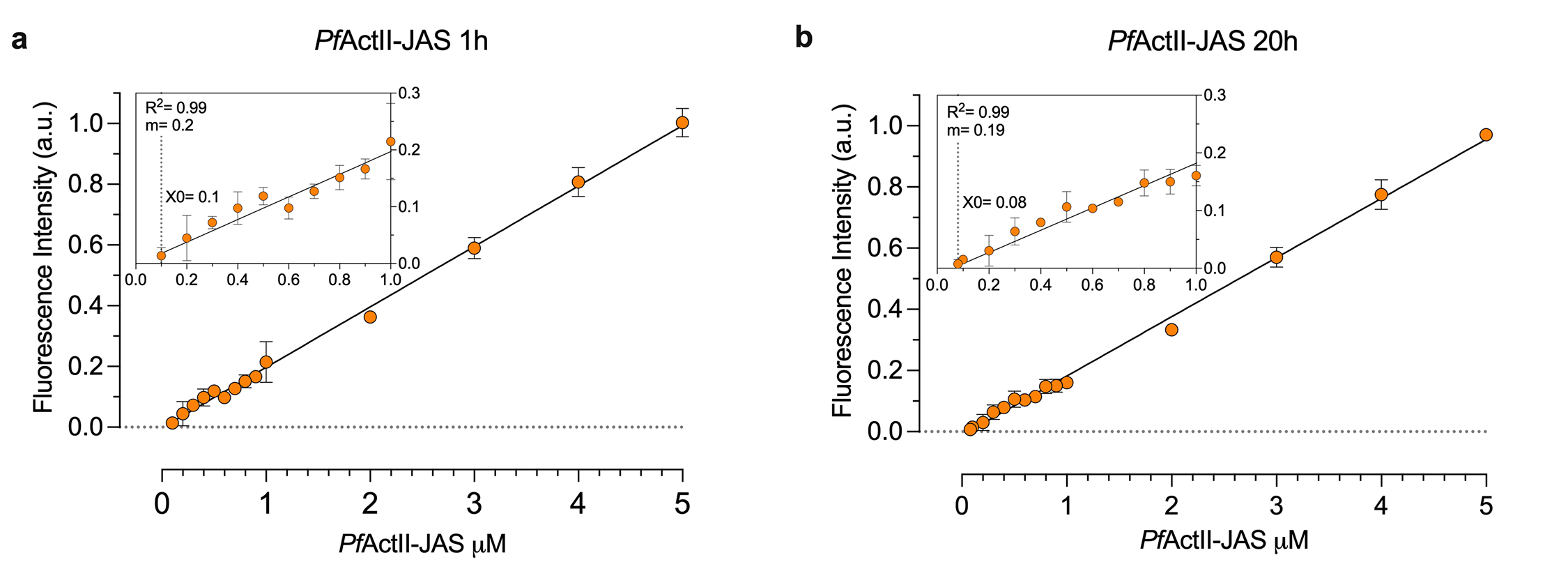

Supplement: S17 Fig — a. Samples after 1 h of incubation at 4°C. b. Samples after 20 h of incubation at 4°C. The data in both panels fit in a linear regression equation. X0 represents the critical concentration. The lower concentration points are magnified on the left. Error bars represent the standard deviation, a.u. = arbitrary units. nb = biological replicates with 3 technical replicates per experiment. Raw data related to this figure can be found in S5 Data. (TIF) [file ppat.1011174.s020.tif]
