# Supplementary material for: Structure and function of Plasmodium actin II in the parasite mosquito stages
Source: PLoS Pathog. 2023 Mar 6;19(3):e1011174. doi: 10.1371/journal.ppat.1011174 (PMC10019781; doi:10.1371/journal.ppat.1011174)
Supplement: Attachment — Submitted filename: rebuttal.pdf [file ppat.1011174.s033.pdf]

Dear Drs. Higgins, Soldati-Favre, Haldar, and Malim,

we thank you and the reviewers very much for your positive and constructive comments on our manuscript. Please find below in blue our detailed replies to the reviewer comments. I have also included both tracked and "clean" versions of the revised manuscript and high-resolution figure files, which have been checked using your online tool, as well as separate supporting file documents. All raw data used to generate the graphs in the figures and the structure validation reports have also been included as supporting files.

I thank you again for your time and hope you now find our manuscript sufficiently improved for publication in PLoS Pathogens.

Sincerely,

Inari Kursula, on behalf of all authors

Reviewer's Responses to Questions

Part I - Summary
Please use this section to discuss strengths/weaknesses of study, novelty/significance, general execution and scholarship.

Reviewer #1: In this excellent paper the authors combine high resolution structural analysis with reverse genetics to investigate actin II in Plasmodium parasites. Plasmodium has a ubiquitously expressed actin I and a divergent actin II expressed in the mosquito stages. Both actins are essential for the parasite and of high interest to understand actin biology across eukaryotic life. While structures and several transgenic lines expressing mutant versions are available for actin I, the current paper investigates both for actin II.

Reviewer #2: The authors reported spatio-temporal distribution in cells, as well as biochemistry and structure, of Plasmodium actin II. The detailed features of Plasmodium actin II achieved by well-organized experimental procedures should be of benefit to a wide range of researchers in the field of pathogens and cytoskeletons. I believe this manuscript deserves publication in PLOS Pathogens.

Reviewer #3: The manuscript by Lopez et al presents a detailed structural and biophysical characterisation of actin II from Plasmodium falciparum.

They start by showing a different expression pattern within the Plasmodium life cycle for actin II compared with actin I, with most expression in gametocytes, with actin mostly found in the nucleus, unlike actin I. This indicates a likelihood of divergent functions for actin II vs actin I. This seems convincing and is largely well done, albeit with challenges to understand figure 1 due to lack of labelling.

They next determine the structure of actin II filaments using cryo-EM methods. This is well done and is accompanied by an extensive discussion and comparison with other actins, including Plasmodium actin I.

They next conduct an experiment in which they introduce a stabilised form of

actin I into the actin II locus and show that it does not complement. I was not sure about the significance of this experiment, but the authors make an appropriately conservative interpretation that other features of actin II are also important, which seems sensible.

The authors then identify methylation of actin II and so that the methylated residue is important for function, and they explore the polymerisation behaviour and dynamics of actin II.

In summary, this is a useful manuscript, which presents the first structural and detailed biophysical characterisation of Plasmodium actin II and identifies features, in expression pattern, localisation and biophysics through which it differs from actin I. The manuscript doesn't identify a novel function of actin II, but it is still a valuable contribution to the field and worthy of publication.

R: We sincerely thank all the reviewers for appreciating the importance and quality of our work and for the constructive comments that help improve our manuscript. Please find detailed responses to all points raised below. We hope to have addressed your concerns in the revised manuscript.

Part II – Major Issues: Key Experiments Required for Acceptance
Please use this section to detail the key new experiments or modifications of existing experiments that should be absolutely required to validate study conclusions.

Generally, there should be no more than 3 such required experiments or major modifications for a "Major Revision" recommendation. If more than 3 experiments are necessary to validate the study conclusions, then you are encouraged to recommend "Reject".

Reviewer #1: no experiments required, there is great scope for future work, but please elaborate/speculate in discussion:
could there be a gene expression difference in the H73 mutant? If not, how else can the "late" phenotype in oocysts be explained?

R: In our previous work, we have validated the approach for replacing the *actin II orf* with either mutant *actin II* or *actin I* (Andreadaki et al. Cell Microbiol 2014, PMID: 24471657); the replacement is carried out in the *actin II* locus while retaining the 5' and 3' control regions intact. In that study, we verified that, in the replacement mutants, the gene was transcribed at comparable levels to the WT locus. We have carried out several studies, which have revealed a second function of actin II in the zygote or ookinete stage which manifests in the oocysts; the mutant oocysts do not develop normally with aberrant DNA replication and reduced size. (Andreadaki et al. Cell Microbiol 2014, PMID: 24471657; Andreadaki et al. Parasitol Res 2016, PMID: 27225004; Vahokoski et al. PLoS Pathog 2014, PMID: 24743229). These data are summed up in the Introduction (lines 90-100). Our manuscript adds important new data to shed light on this second function, as we determined that the protein is present in the zygote stage (western blot Fig 1a, localization in Fig 1 d,e). In the manuscript, this is discussed on lines 530-558. Since little is known about the zygote stage, it is not possible to pinpoint a specific function of actin II in this stage, but we speculate that subtle changes in

filament properties interfere with the molecular function of actin II.

Reviewer #2: (No Response)

Reviewer #3: (No Response)

Part III – Minor Issues: Editorial and Data Presentation Modifications
Please use this section for editorial suggestions as well as relatively minor modifications of existing data that would enhance clarity.

Reviewer #1: I hope the following helps to improve an already phenomenal paper:

37: "We show… zygotes…" – maybe better to write "We confirm expression and how function", also: I would suggest to add function during "oocyst stage"

R: We have reworded the Abstract as suggested.

44/105/…: I understand that paper such that actin II is expressed in zygote but not oocysts, but that there is a functional deficiency in oocysts and not zygotes. If correct, please modify to state "oocyst" instead of "zygote" when talking about function.

R: We changed "zygote" to "oocyst" in the Abstract (lines 35 and 45) and Significance statement (line 56). In the Introduction, "zygote" relates to expression not function (lines 58, 94, 113).

79/80: modify to say "development, motility and invasion" and cite the work by Douglas (ie ref 49 and Yee et al., Plos Path 2022) to qualify "intensely studied" and the work by Das et al BMC Bio 2017 to qualify "development and invasion".

R: We have modified the sentence as suggested and included the suggested references (now line 87).

182: I don't see how localization offers new insight into function, please rewrite or delete

R: We have omitted the latter part of the sentence, as suggested (now line 191).

193: add a sentence on how you purified actin II

R: We have added a short mention that we used recombinantly produced actin II in this paragraph (now line 200). Details of the purification are given in the Materials and methods.

208 and onwards: please use sensible digits, e.g 167 instead of 166,9°

R: We have rounded up the helical twist angles, as suggested (now lines 220-222).

313: delete first "and"

R: Thank you, the surplus "and" has been deleted (now line 335).

646: not sure "filament like structures" is the best way to describe the accumulations

R: We have reworded this sentence (now line 674).

Reviewer #2: 1 It is difficult to follow overall structural difference among the subunits of PfActII, PfActI and alpha-actin. A superposed diagram of one subunit of each species such as Fig. S16 would be helpful.

R: The overall structures of these three actins are very similar. Therefore, we believe that an overall superposition would not be very informative. For this reason, we had prepared figures S3 and S6 as well as movie S1, which highlight the conserved and non-conserved regions (S3 Fig and S1 Movie) as well as the differences in the twist angles of these actins (S6 Fig). Specific structural differences at the monomer level have been described in detail in Vahokoski et al. PLoS Pathog 2014, PMID: 24743229.

2 Fig. 1 presented spatio-temporal distribution of actin II in the cell. I agree with the authors that the rod-like distribution imply filament formation. However, staining actin II with rhodamine phalloidin or lifeact, which binds to the actin filaments, will give more clarity.

R: In our previous work, we have also looked at actin I in the gametocyte, using a specific antibody against this isoform (Curra et al. Exp Parasitol 2017, PMID: 28803903). The protein is highly expressed and found in the cytoplasm with no distinct localization. Sedimentation experiments showed that actin I forms filaments in this stage. Mass spectrometry of extracts from gametocytes suggested that actin I is present in at least six fold excess compared to actin II (unpublished data). Therefore, any signal from filamentous actin II would be very difficult to separate from the signal of actin I. Furthermore, phalloidin and LifeAct do not stain Plasmodium actins, as discussed in Das et al. Cell Microbiol 2021, PMID: 33885206, although recently the use of a genetically encoded Chromobody has been used to visualize actin I in *Plasmodium* (Yee et al. PLoS Pathog 2022, PMID: 35998188; Stortz et al. eLife 2019, PMID: 31322501). At this time, these experiments would be very difficult to perform due to limited time and resources, and we sincerely hope that such experiments could be left for follow-up work.

3 Line 269: "(Fig 4a-5c)" should be "(Fig 4a-4c)".

R: Thank you, this has been corrected together with other

improvements to the referencing to the figures in this paragraph (now from line 282).

4 Lines 375-376: "Under polymerizing conditions, actin II was distributed into three populations with rH of 8.1, 43, and 410 nm, with volume percentage contributions of 51, 33, and 17%." I believe the authors could observe images corresponding to the fraction with rH of 43 nm by electron microscopy. The authors should report it. The state of this fraction might be largely different from the filament and it might cause the two linear slopes in Figs 8c and d.

R: We show representative electron micrographs of both actin I and actin II in Fig 7e. The observed sizes in DLS (Fig 7c) and in the micrographs in Fig 7e are not directly comparable, and we have not observed in EM species that we think would correspond to the 43 nm peak. We believe that this is partly due to the differences in the methods (e.g. different protein concentrations, handling of the samples during negative staining, etc.) and partly due to the possibly transient nature of these structures. We think the DLS results are mainly useful for comparing the size distributions in the different samples (different actins and the actins with or without JAS). The trend we see is that both Plasmodium actins form smaller structures than muscle actin, that actin II forms larger entities than actin I, and that JAS shifts the samples towards larger species. This trend correlates with what we see in the electron micrographs, although the sizes do not.

5 Lines 550-551: "In actin II, fragmentation could be mediated in vivo by other mechanisms, such as ABPs or the intracellular environment." The authors should cite papers showing evidences for fragmentation of actin II filaments in vivo.

R: We apologize for this sentence being unclear. We meant to present this as a hypothesis, as we do not observe the A loop movement in actin II. We have reworded (now lines 577-578).

6 Lines 1321-1322: I could not find "green star" in Fig S11a.

R: The legends for the panels in this figure were unfortunately swapped. This has now been fixed and the legend also otherwise improved.

Reviewer #3: • A number of the figures are not particularly well labelled or explained and would benefit from work to make the manuscript more accessible to a reader.
• Please adapt the labelling of Figure 1a to make it easier to read – for example label the lanes with their contents rather than just numbers which require the legend to interpret.

R: We have included self-explanatory labels to Fig 1a and modified the legend accordingly.

• Please rewrite the legend to Figure 1b-e, which is very confusing and unclear.

Which images have just the secondary control? What are c and e, which have no labelling at all? The figure needs labelling properly.

R: We have improved the legend. The secondary controls were not included in the figure, and we have changed the text to: Controls with only the secondary antibody were included in the experiment; these had no signal (not shown). (b-e) has been changed to (b,c). c and e, as explained in the legend, are the single slices of the z-stacks from b and d, respectively.

• *The entire section from lines 207-231 is only describing supplementary figures. If important enough for a full section of the text, then some of the data could be shown in panels in Figure 2 in the main manuscript.*

R: These data are in our opinion best visualized as movies rather than still images. Therefore, the text references only supplemental files. We feel that the manuscript already has many and rather large figures, and therefore, we would prefer to keep the information in Figures S6 and S7 in the supplement.

• *Figure 3 – these isn't a good match to the text section of lines 244-257. For example, GgAct isn't described in the text. The same is true when comparing lines 262-273 with Figure 4. Please join up the figures and the text.*

R: We have improved the referencing to Figs. 3 and 4 in the corresponding text (now lines 261-293). GgAct in both figures represents "canonical" actins. This is now hopefully clear from the references to the panels.

• *Figure 5C – what is the difference between the central and right-hand columns?*

R: These are two representative examples of mutant oocysts, which is now more clearly mentioned in the legend. The oocysts come in different sizes in the mutant, some containing DNA and others not.

• *Figure 7B – why has the PfAcI not been studied at 100k?*

R: We have previously reported similar data for actin I using both 100000 and 435000 g (Kumpula et al. Sci Rep 2017, PMID: 28939886). Similarly to actin II here, a smaller fraction of actin I was pelleted at 100000 g than at 435000 g. As this had already been reported, and actin I served more as a control in this set of experiments, we only used the higher g force for actin I here.

• *Figure 7C – label with arrows the species described in the text?*

R: We have now indicated the 8.1, 43, and 410 nm peaks with arrows in the graph and updated the legend accordingly.

• *Figure 8 – why is actin I studied after 1h and atcin II after 20h?*

R: In fact, both 1 h and 20 h polymerization times are presented for both actins in figure 8. Panels a and b show 1 and 20 h, respectively, for actin I and panels c and d the same for actin II.

• *Finally, have the coordinates and cryo-EM data been deposited. There were no validation reports provided. The manuscript shouldn't be accepted without these being provided for the reviewers to check.*

R: The structures have been deposited and have the PDB codes 8CCN and 8CCO The validation reports are attached.
